# ESBL carriage, implementation variations of Dutch guidelines

**DOI:** 10.1186/1753-6561-5-S6-P137

**Published:** 2011-06-29

**Authors:** G Terlaak-Harbers, F Loeffen, L Ummels, J Hopman

**Affiliations:** 1 Medical Microbiology, Radboud University Nijmegen Medical Centre, Nijmegen, Netherlands

## Introduction / objectives

In 2005, the Dutch Working party Infection Prevention (WIP) published, its “Measures to prevent transmission of highly resistant microorganisms (HRMO)” guidelines. These guidelines provide strict definitions for the indications and measures that have to be taken in patients with HRMO, including for cases of ESBL carriage. In this study we aim to evaluate the implementation of these guidelines with regard to ESBL.

## Methods

In September 2010 a questionnaire with 10 items has been sent to 90 Dutch hospitals (8 teaching hospitals, 82 non-teaching hospitals). Matters addressed were: origin of culture site, notification of infection control professionals, type and duration of isolation at (re-) admittance and type of department (ICU and non-ICU).

## Results

Response rate to the questionnaire was 69% (7 teaching hospitals, 55 non-teaching hospitals). In 26/62 hospitals, Dutch culture recommendations for detection of ESBL carriage, namely throat and anal swab/faeces cultures, were not followed. Notification of infection control professional after ESBL detection occurred in 59/62 hospitals, at re-admittance in 31/62 hospitals. In 3/62 hospitals infection control measures were carried out based on individual risk assessment in every subsequent patient. Sixty percent of the non-teaching hospitals (33/55) used droplet isolation in case of respiratory ESBL isolates. In teaching hospitals this is the case in 2/7 institutes. At re-admittance the duration of isolation ranges from 2 months until the end of admittance.

## Conclusion

In spite of clear ESBL carriage guidelines, major differences exist between hospitals concerning the implementation of these guidelines.

The main differences were: origin of culture site, notification after re-admittance and type of isolation measures.

## Disclosure of interest

None declared.

